# New Insights into the Role of Polybromo-1 in Prostate Cancer

**DOI:** 10.3390/ijms20122852

**Published:** 2019-06-12

**Authors:** Sara T. S. Mota, Lara Vecchi, Mariana A. P. Zóia, Fabrícia M. Oliveira, Douglas A. Alves, Bruno C. Dornelas, Stephania M. Bezerra, Victor P. Andrade, Yara C. P. Maia, Adriana F. Neves, Luiz Ricardo Goulart, Thaise G. Araújo

**Affiliations:** 1 Laboratory of Genetics and Biotechnology, Institute of Biotechnology, Federal University of Uberlandia, Patos de Minas-MG 387400-128, Brazil; saratsm.s@hotmail.com (S.T.S.M.); douglasalexsanderptu@hotmail.com (D.A.A.); 2 Laboratory of Nanobiotechnology, Institute of Biotechnology, Federal University of Uberlandia, Uberlandia-MG 38400-902, Brazil; marianazoia@hotmail.com (M.A.P.Z.); lrgoulart@ufu.br (L.R.G.); 3 Faculty of Mathematics, Federal University of Uberlandia, Patos de Minas-MG 387400-128, Brazil; fabricia@ufu.br; 4 Pathology Division, Internal Medicine, University Hospital, Federal University of Uberlandia, Uberlandia-MG 38400-902, Brazil; dornelasbruno@hotmail.com; 5 AC Camargo Cancer Hospital, São Paulo-SP 01509-010, Brazil; stephania.bezerra@accamargo.org.br (S.M.B.); victor.andrade@accamargo.org.br (V.P.A.); 6 Medical Faculty, Federal University of Uberlandia, Uberlandia-MG 38400-902, Brazil; yaracpmaia@gmail.com; 7 Laboratory of Molecular Biology, Federal University of Goias-GO, Goiânia-GO 75704-020, Brazil; neves.af@gmail.com; 8 University of California Davis, Department of Medical Microbiology and Immunology, Davis, CA 95616, USA

**Keywords:** Polybromo-1, SWI/SNF, prostate cancer, Gleason score, epithelial-mesenchymal transition

## Abstract

The human protein Polybromo-1 (PBMR1/BAF180) is a component of the SWI/SNF chromatin-remodeling complex that has been reported to be deregulated in tumors. However, its role in prostate cancer (PCa) is largely unknown. In this study, we described the PBRM1 transcriptional levels and the protein expression/localization in tissues of PCa patients and in prostatic cell lines. Increased *PBRM1* mRNA levels were found in PCa samples, when compared to benign disease, and were correlated with higher Gleason score. We also verified that only the nuclear localization of PBRM1 protein is correlated with a more aggressive disease and high Prostate-Specific Antigen (PSA) levels in tissue microarrays. Intriguing expression patterns of mRNA and protein were identified in the cell lines. Although PBRM1 protein was restricted to the nuclei, in tumor cell lines in non-neoplastic cells, it was also present in vesicular-like structures that were dispersed within the cytoplasm. We knocked-down PBRM1 in the castration-resistant PCa (CRPC) cell line PC-3 and we verified that PBRM1 promotes the expression of several markers of aggressiveness, including EpCAM, TGF-β, and N-Cadherin. Therefore, our data supported the hypothesis that PBRM1 displays a pivotal role in the promotion and maintenance of the malignant behavior of PCa, especially in CRPC.

## 1. Introduction

Prostate cancer (PCa) is one of the most common cancers worldwide [1], which is characterized by a heterogeneous, multifactorial, and multifocal disease, with few clear histopathological subtypes [2]. Radiation therapy and surgery are the gold standard treatments for the early stages of PCa. However, patients that display an advanced or metastatic disease are also treated with the androgen-deprivation therapy (ADT) that consists by surgical or pharmacological castration [3]. Although ADT reduces the progression of PCa and significantly decreases the number of deaths [4], the insurgence of castration-resistant PCa (CRPC) [5] results in a high rate of treatment failure [6]. CRPCs also develop thanks to the epithelial-mesenchymal transition (EMT), a process in which epithelial cells change to a highly metastatic fibroblast-like phenotype [7]. Therefore, ADT can induce the EMT process that, in turn, may lead to an increase in cancer stem cells (CSCs) [8]. These events result in the aggressiveness and lethally of CRPC [9]. In this context, attempts to identify molecular alterations that influence oncogenic pathways are valuable in understanding PCa and guiding diagnosis, prognosis, and therapeutic strategies [10]. Cancer is driven by a number of multiple events and special attention has been devoted to the chromatin regulation and epigenetic processes [11]. ATP-dependent chromatin-remodeling complexes permit the packaging and unpackaging of DNA that, in a neoplastic environment, might suppress or trigger cancer development [12]. However, it is noteworthy that chromatin regulators have rich and biologically diverse regulatory roles through still unknown mechanisms [13].

The SWI/SNF (SWItch/Sucrose Non-Fermentable) family comprises chromatin-remodeling complexes that use the energy of ATP hydrolysis to alter the histone–DNA interactions and to modulate transcription. In humans, two different SWI/SNF complexes exist: the BRG1- or hBRM-associated factors (SWI/SNFA or BAF) and the SWI/SNFB or polybromo-associated BAF (PBAF). The specificities of each complex are due to their exclusive subunits. The AT-rich interactive domain-containing protein 1A (ARID1A; also known as BAF250A or SMARCF1) and ARID1B subunits are mutually exclusive and unique for BAF. The subclass PBAF possesses BAF180 (also known as PBRM1), BAF200, and bromodomain-containing 7 (BRD7) [14,15,16].

PBAF coordinates multiple central functions in chromatin remodeling; it targets DNA sites, recruits specific effector proteins, and alters the histone-DNA interactions [12,14]. Among its proteins, the human protein Polybromo-1 (PBMR1/BAF180) contains 1689 amino acids and six tandem bromodomains (Brds), two bromo-adjacent homology domains (Bah), and a high-mobility group (Hmg). Each one of these Brds is made up of about 100 amino acids and it binds lysine residues modified by acetylation. Bah domains are involved in protein-protein interactions and the Hmg domain binds nucleosomal DNA [17]. While it is clear that PBRM1 confers specificity to PBAF, it is currently unclear how it drives tumorigenesis. The existing knowledge regarding its function includes its role in the control of cell cycle [18] and the transcription of estrogen-responsive genes [19]; promotion of genomic stability [20]; regulation of cellular senescence [21], apoptosis [22], centromeric cohesion [20], and ubiquitination events [23]; and, prevention of aneuploidy [20]. Alterations in the *PBRM1* gene have been described in approximately 2–14% of breast cancers and 40% of renal cancers [19,24,25]. However, this PBAF subunit seems to act differentially in each cancer type [19,22,26,27,28] and its role in PCa has not yet been described. Although other SWI/SNF subunits have already been associated to androgen regulation, helping in diagnosis and prognosis of the disease [29,30,31,32,33]; information about the role of PBRM1 in PCa are still missing.

In this study, we hypothesized that PBRM1 has a tissue-specific and instructive role in PCa. Unraveling the oncogenic mechanisms behind chromatin remodelers highlights important gaps in our knowledge about tumors and it may suggest potential targets for cancer management. PCa biological aggressiveness displays strong molecular variations. Thus, we propose that new insights in PBRM1 may elucidate regulatory events, which may define the clinical outcome. To test this notion, we evaluated the transcriptional and translational levels of PBRM1 in tissues of patients with PCa and benign prostatic hyperplasia (BPH), and in four prostate lineages, including one non-neoplastic (RWPE-1), one androgen-responsive (LNCaP), and two CRPC cell lines (PC-3 and DU-145). We described that the PBRM1 transcriptional and protein levels are higher in patients with PCa, when compared to those with BPH, and correlate with tumor aggressiveness. Besides its standard nuclear localization, we found that PBRM1 can also localize in vesicular-like structures that are dispersed in the cytoplasm of PCa cells. Finally, by knocking down PBRM1 in CRPC cells, we demonstrated its involvement in the EMT process and cell aggressiveness. Our results shed light on the molecular behavior of PBRM1 in PCa. In particular, our findings strive to understand the transcriptional consequences of alterations in chromatin-remodeling complexes and support the notion that PBRM1 plays a critical role in PCa.

## 2. Results

One of the previous findings that motivated this study is that PBRM1 can be found to be differentially regulated in cancer. Given the central role of PBRM1 in oncogenesis, and the lack of information regarding its behavior in PCa, we conducted experiments to elucidate the relevance of PBRM1 as a putative cancer driver in PCa. With this purpose, we started to analyze the expression of PBRM1, both at the transcriptional and translational levels and performed knockdown experiments aimed at understanding its role in CRPC. Consequently, we found that PBRM1 is increased at both transcriptional and translational levels in PCa and correlates with the aggressiveness of the disease. By using a PBRM1 knock down CRPC cell line (PC3 shPBRM1), we found that PBRM1 regulates the expression of EMT and CSC markers, thus enhancing PCa aggressiveness. Therefore, our data supported the hypothesis that the PBRM1, a unique component of the PBAF complex, is important in prostate malignant transformation and aggressiveness.

### 2.1. PBRM1 Expression in PCa Patients

A total of 40 patients were included in this study, and 27 (67.5%) of them had PCa and 13 (32.5%) had BPH (Table 1). Patients’ age did not differ between both groups. The mean Prostate-Specific Antigen (PSA) levels of patients with PCa and with BPH were 9.57 ng/mL and 8.57 ng/mL, respectively. Although PSA levels are routinely used as a diagnostic marker [34,35], there was no statistical difference between PCa and BPH.

We subsequently quantified the mRNA levels of the *PBRM1* gene in tissue samples of PCa and BPH patients (Table 2). We observed that *PBRM1* was 9.8-fold more expressed in PCa tissues when compared to BPH tissues through the Mann–Whitney test (MW). Regarding the tissue samples of PCa patients, the mRNA levels were higher in patients with Gleason score ≥ 7 (Table 2, MW *p* = 0.04). Therefore, we performed a categorization of *PBRM1* expression in PCa and BPH patients based on median value of mRNA levels, in order to compare the differences in transcript levels depending on their clinical and biochemical data. We have obtained a significant odds ratio of 8.33-fold in the PCa patients with Gleason score ≥ 7 (Table 2, OR = 8.33, *p* = 0.02). However, the expression of the *PRRM1* gene did not show differences upon BPH patients.

### 2.2. PBRM1 Protein is Expressed in Nuclei, Cytoplasm and Membrane of PCa Tissues

**Figure 1:**
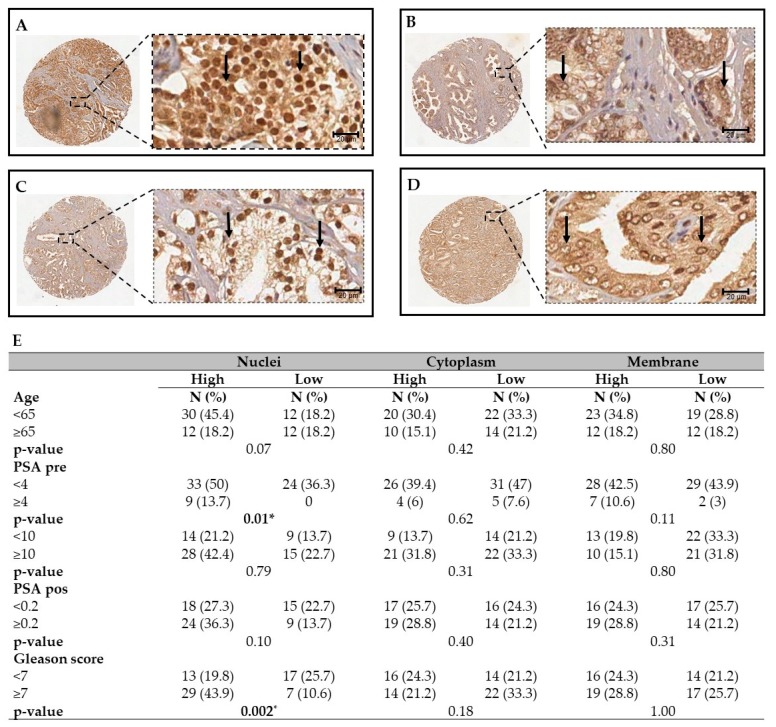
Immunohistochemistry of PBRM1 in tissue microarrays (TMAs) containing prostate cancer tissues. The figure shows representative images of PCa patient-derived tissue microarrays (TMA). (**A**) PCa sample displaying a high Gleason score showing high intensity of total PBRM1 staining. (**B**) PCa sample displaying a low Gleason score sample showing low intensity of total PBRM1 staining. (**C**) PCa sample displaying a high Gleason score and showing a high nuclear and low cytoplasmic intensity of PBRM1 staining. (**D**) PCa sample displaying a low Gleason score showing low nuclear and high cytoplasmic/membranous intensity of PBRM1 staining. Arrows indicate the nuclear localization of PBRM1. (**E**) The table shows the correlation of PBRM1 with histopathological parameters of patients. * *p* < 0.05. PSA: Prostate-Specific Antigen. Scale bar = 20 μm and 20× magnification.

PBRM1 expression was also evaluated by immunohistochemistry in tissue microarrays (TMAs) containing 66 samples from patients with PCa. PBRM1 protein was detected, not only in nuclei, but also in the cytoplasm and membrane (Figure 1A–D). The samples were further categorized by a pathologist, based on the intensity of staining (high and low) and analyzed by Fisher’s exact test. High nuclear levels of PBRM1 were detected in patients with PSA ≥ 10 ng/mL (Figure 1E; *p* = 0.01) and with poorly differentiated tumors that presented a Gleason score ≥ 7 (Figure 1E; *p* = 0.002). Cytoplasmic and membrane staining did not show any significant correlation (Figure 1E). Taken together, these results indicate that a high expression of both PBRM1 mRNA and protein correlate with the aggressiveness of PCa, which therefore suggested that PBRM1 expression could be used as a negative prognostic factor for these tumors.

### 2.3. PBRM1 Expression in Prostate Cell Lines

**Figure 2:**
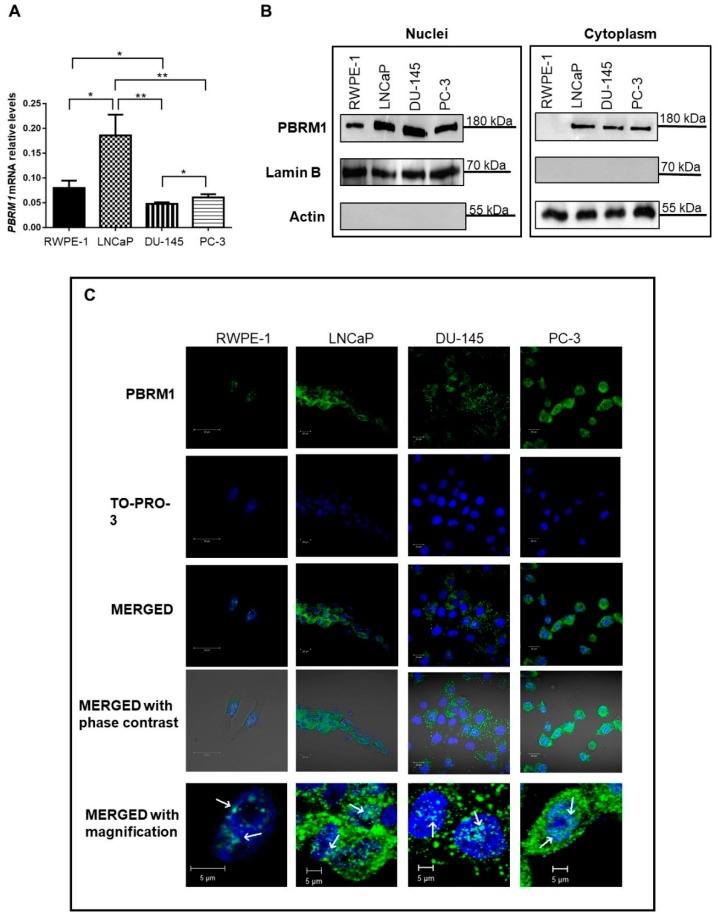
Expression of mRNA levels of *PBRM1* gene and localization of PBRM1 protein in the prostatic cell lines RWPE-1, LNCaP, DU-145, and PC-3. (**A**) mRNA relative levels of *PBRM1* gene in RWPE-1, LNCaP, PC-3, and DU-145 cell lines. * *p* < 0.05; ** *p* < 0.01 (**B**) The cropped Western blotting of PBRM1 expression in membrane, cytoplasm, and nuclear fractions of RWPE-1, LNCaP, DU-145 e PC-3 cells. Full length PBRM1 (180 kDa) was detected. Actin and Lamin B were used as loading controls of cytoplasmic and nuclear extracts, respectively, and as controls of proper cell fractionation. (**C**) Immunofluorescent localization of PBRM1 was analyzed by staining cells with anti-PBRM1 (1:50, green), whereas nuclei were counter-stained with TO-PRO-3® (1:500, blue). Merged images (light blue) confirmed nuclear localization of PBRM1 in all cell lines. Cytoplasmic localization was only detected in prostate cancer cells. An intensive vesicular-like staining was observed in the cytoplasm of LNCaP, PC-3, and DU-145 cells. Arrows indicate nuclear localization of PBRM1. Three independent experiments were performed. Scale bar = 20 μm and 5 μm (magnification).

Assays using prostate cell lines were performed to better understand PBRM1 behavior. We first analyzed the transcriptional levels of *PBRM1* in three PCa cell lines, an androgen-sensitive PCa cell line, LNCaP, and two CRPC cell lines, PC-3 and DU-145. The non-tumorigenic prostate cell line RWPE-1 was used as the control. Higher mRNA levels of *PBRM1* were detected in LNCaP cells when compared to the other cell lines (Figure 2A). The *PBRM1* mRNA levels were 2.3, 3.9, and 3.1-fold higher in LNCaP when compared to RWPE-1 (t test, *p* = 0.014), DU-145 (t test, *p* = 0.005), and PC-3 cells (t test, *p* = 0.007), respectively. On the contrary, the two CRPC cell lines, displayed lower levels of *PBRM1* mRNA either when compared to LNCaP or RWPE-1 cells (Figure 2A).

Western blotting and immunofluorescence analyses revealed that PBRM1 could be found in different cellular compartments (Figure 2B,C). In RWPE-1 cells, PBRM1 was only identified in nuclei; whereas, in all the PCa cell lines, either androgen-responsive (LNCaP) or CRPC (DU-145 and PC-3), the protein was detected in nuclei and cytoplasm. Moreover, in all the three PCa cell lines, we observed that the cytoplasmic fraction of PBRM1 displayed a vesicular pattern. Taken together, these results suggest that PBRM1 localization and, specifically, both its nuclear and cytoplasmic localizations, may be related to prostate tumorigenesis.

### 2.4. Decreased Expression of PBRM1 Hampers PCa Aggressiveness

**Figure 3:**
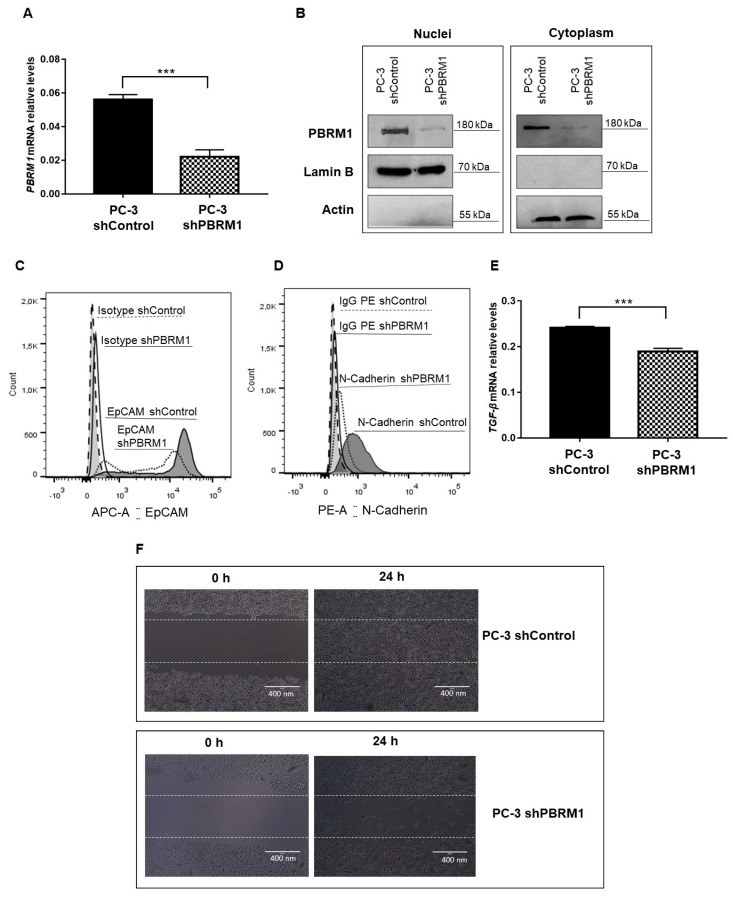
Analysis of PBRM1-downstream cellular events in PC-3 cells. (**A**) mRNA relative levels of *PBRM1* gene in PC-3 cell line stably knocked down for PBRM1 (PC-3 shPBRM1) compared to a PC-3 cell line stably expressing a control shRNA (PC-3 shControl) (**B**) The cropped Western blotting of PBRM1 expression in membrane/cytoplasm (M + C) and nuclei (N) fractions of PC-3 shPBRM1 and PC-3 shControl cells. Full length PBRM1 (180 kDa) were detected. Actin and Lamin B were used as loading controls of cytoplasmic and nuclear extracts and as controls of proper cell fractionation. (**C**) EpCAM and (**D**) N-Cadherin were analyzed in PC-3 shPBRM1 and compared to PC-3 shControl cells by flow cytometry. (**E**) mRNA relative levels of *TGF-β* gene in PC-3 shPBRM1 and PC-3 shControl cells. (**F**) Migration potential was assessed by wound healing assay. PC-3 shControl and PC-3 shPBRM1 cells were plated, scratched with cell scraper, and photographed by phase-contrast microscopy. Representative images, showing cells migrated at 0 h and after 24 h. *** *p* < 0.001 is significant. Three independent experiments were performed. Scale bar = 400 nm.

In order to describe the role of PBRM1 in PCa oncogenesis and progression, we established a PC-3 cell line stably knocked down for PBRM1 (PC-3 shPBRM1) and compared to a PC-3 cell line stably expressing a control shRNA (PC-3 shControl). We analyzed both *PBRM1* mRNA levels by qRT-PCR and PBRM1 protein levels by Western blotting, which confirmed the success of silencing experiments (Figure 3A,B). Flow cytometry assay was performed to evaluate the effect of PBRM1 on the expression of several markers that were commonly studied to describe the EMT process, including E-Cadherin, Vimentin, and N-Cadherin [36]. The expression of the CSC marker, EpCAM [37], was also measured by flow cytometry. We found that the expression of EpCAM and N-Cadherin decreased in PC-3 shPBRM1 (Figure 3C,D). On the contrary, the expression of Vimentin and E-Cadherin did not show differences upon PBRM1 knock down in the PC-3 cells. We also quantified the mRNA expression of *TGF-β*, a cytokine that has been also involved in the EMT process, by qRT-PCR [38]. *TGF-β* transcripts also decreased in PC-3 shPBRM1 (Figure 3C). Finally, by wound healing assay, PC-3 shPBRM1 cells displayed decreased migration properties when compared to their control cells (PC-3 shControl).

These results agree with the above findings in which PCa patients with a higher histological grading showed increased expression of PBRM1, indicating a tumor promoting function of PBRM1.

## 3. Discussion

PCa represents a public health concern. The major clinical problem relies on the management of patients that stop to respond to ADT upon the development of CRPC. CRPC is a lethal disease and the median survival of the patients is about 15–36 months [39]. CRPC is characterized by the sustained activation of the androgen receptor (AR) axis [40]. Although the etiology remains unknown, some evidences indicate the essential role of epigenetic modulation in CRPC [41,42].

The chromatin remodeler proteins regulate the accessibility of transcriptional factors to the DNA, thus controlling the gene expression and biological functions [43]. An aberrant expression or an aberrant modulation of chromatin remodelers allows for the cancer cell to reprogram its genome, thus resulting in the maintenance of the malignant phenotype [44]. Hence, the understanding of chromatin remodeler function is essential in order to unravel the mechanisms underlying the tumor promotion and, importantly, how different pathways are deregulated for progression and aggressiveness.

PBRM1 is a key component of the SWI/SNF chromatin-remodeling complex and it is widely considered as an oncosuppressor gene in various cancer types by virtue of its cell cycle regulation activity [21]. Although much effort has been devoted to understanding the PBRM1 function in renal cancer [25,45,46,47,48,49,50], only incipient information regarding its role in PCa is available. To our knowledge, this is the first report that analyzed the transcriptional and protein expression levels of PBRM1 in patients with PCa and in prostatic cell lines and investigated putative molecular mechanisms that are modulated by PBRM1. In this research, we sought to systematically evaluate the correlation between PBRM1 and PCa occurrence and progression. Our findings provided complementary means of assessing the behavior of this protein in non-neoplastic prostate cells, androgen-sensitive PCa cells, and CRPC cells. Remarkably, in this manuscript, we succeed in demonstrating that PBRM1 expression is higher at both the transcriptional and protein levels in PCa. By means of its correlation with cell aggressiveness parameters, and according to our results of knockdown experiments, we suggest that PBRM1 represents a negative prognostic factor in PCa.

In the first part of this manuscript, we analyzed patients’ samples and described that the transcriptional levels of *PBRM1* were significantly higher in PCa tissues when compared to BPH tissues. Hence, *PBRM1* transcript analysis can discriminate the benign samples from those with PCa. Notably, the transcriptional levels of *PBRM1* correlated with Gleason score. By analyzing TMAs containing patients’ PCa samples, we identified PBRM1 expression in nuclei, cytoplasm, and membrane of cancer cells. Interestingly, we observed that exclusively the nuclear localization of PBRM1 also correlated with PCa aggressiveness (higher Gleason score). Gleason score is an important histopathological analysis [47,51]; and, the higher the score, the more undifferentiated the lesion, which increases the chances of invasion and metastasis [52]. Markers that are correlated with Gleason score are generally associated with a worse prognosis and they may be potential targets for promising therapies [53,54]. Our results agree with a previous work that demonstrated that PBRM1 expression is a negative prognostic factor in renal carcinoma [47]. Moreover, when considering the role of PBRM1 in modulating gene expression, our findings could indicate the regulation of different pathways that are driven by PBRM1 in promoting oncogene transcription in PCa.

Therefore, we have analyzed the transcriptional levels of *PBRM1* in prostate cells and observed that higher mRNA levels were detected in LNCaP cells. Regarding the PBRM1 protein, we found that it was uniquely localized in the nuclear compartment of RWPE-1 cells. In the three PCa cell lines, it also strongly localized at the cytoplasmic level, demonstrating an intriguing behavior in PCa. Since it is well known that PBRM1 allows the transcription of several genes that are involved in the inhibition of cell cycle [21], its nuclear localization in RWPE-1 cells suggests that, in non-tumor conditions, PBRM1 exerts a tumor suppressor function. PBRM1 localization in both nuclei and vesicular-like structures dispersed within the cytoplasm of all PCa cell lines (LNCaP, PC-3, and DU-145) could indicate that PBRM1 can complex with cytoplasmic transcription factors. Thus, these transcription factors could migrate to nuclei to bind different DNA regions, regulating the expression of genes that are involved in tumor promotion and progression. Eventually, PBRM1 in the cytoplasm could also participate in signaling events not yet known.

The study of PBRM1 expression in prostate cell lines also revealed information regarding PBRM1 protein stability among different subtypes of PCa. We observed that, when compared to the non-tumorigenic cell line, RWPE-1, *PBRM1* transcriptional levels were higher in the androgen–sensitive PCa cell line, LNCaP, and lower in the two CRPC cell lines, PC3 and DU-145. Subsequently, we observed a discrepancy between the transcriptional and protein levels of PBRM1. The PBRM1 protein levels were higher in all the three PCa cell lines when compared to the RWPE-1 cells. Hence, despite the low mRNA levels, the PBRM1 protein accumulated in the two CRPC cell lines, PC3 and DU-145. This finding could indicate that the PBRM1 protein displays a higher stability in CRPC cells contributing to PCa progression. Alternatively, translational rate of *PBRM1* mRNA in CRPC cells may be higher when compared to the LNCap and RWPE-1 cells in order to modulate tumorigenesis.

The malignant behavior of PBRM1 in CRPC cells was confirmed by establishing a PC-3 cell line that was stably knocked down for PBRM1 (PC-3 shPBRM1). As a matter of fact, we confirmed that PBRM1 triggers the EMT process and therefore participates in the maintenance of the aggressive phenotype of CRPC cells. The decreased expression of PBRM1 led to a significant decrease of the EMT markers, N-Cadherin, and TGF-β [38,55]. N-Cadherin is considered to be a crucial protein in the acquisition and maintenance of the aggressive phenotype; it displays a pivotal role in metastasis formation and in the acquisition of resistance to castration [56,57]. TGF-β is a cytokine that promotes invasion and metastasis in the later stages of PCa and, indeed, its signaling increased in CRPCs [58,59]. We also observed a decrease in the CSC marker, EpCAM, which has been previously associated with an increased metastatic potential of PCa cells [37]. We infer that PBRM1 displays a pivotal role in CRPC modulating EMT and CSC markers, which makes it a target for inhibiting PCa aggressiveness.

In this study we succeed in showing the PBRM1 expression levels and its clinical relevance in PCa. Moreover, we described the function of PBRM1 in inducing the aggressiveness of PCa by demonstrating its involvement in the expression of the EMT and CSC markers. The identification of oncogenic pathways that are modulated by PBRM1 could lead to alternative strategies in the treatment and diagnosis of PCa. Moreover, further studies on PBRM1 deregulation and its associated pathways could contribute to better understanding the pathogenesis and aggressiveness of PCa, especially CRPC.

## 4. Materials and Methods

### 4.1. Patients’ Samples

This work was developed at the Laboratory of Nanobiotechnology of the Federal University of Uberlandia (UFU), together with the Urology Service of the University’s Clinical Hospital. All of the experiments were performed in accordance with relevant guidelines and regulations. The study was conducted in accordance with the Declaration of Helsinki. The Ethics Committee of the Institutional Research Board of the Federal University of Uberlandia approved all of the procedures (number 005/2001—02/19/2001). Informed consent form was obtained from each participant.

For transcripts quantification, forty tissue samples were obtained from 27 PCa patients that were submitted to radical prostatectomy and 13 BPH patients submitted to transurethral resection. IMMULITE 1000 System (Siemens Healthcare Diagnostics Inc., Munich, Germany) was used for quantitative detection of PSA levels, when considering normal values between 0 and 4.0 ng/mL. The patients were categorized as high or low PSA by using two different cut-off values: 4 and 10 ng/mL [53]. Eligible men were those who were not submitted to neo-adjuvant chemotherapy, radiation, or hormonal therapy.

### 4.2. Prostate Cell Lines

For this study, four prostate cell lines that were obtained from ATCC were used after authentication through STR analysis: a non-tumorigenic prostate cell line (RWPE-1), an androgen-responsive cell line (LNCaP), and two CRPC cell lines (PC-3 and DU-145). The non-tumorigenic lineage was cultivated in Keratinocyte Serum-Free Medium (KSFM) that was supplemented with 5.0 ng/mL of Epidermal Growth factor (EGF) and 0.05 mg/mL of pituitary extract. The PC-3 and LNCaP cells were maintained in RPMI 1640 medium, and DU-145 cells were cultivated in Dulbecco Modified Eagle Medium (DMEM). All media contained 10% of fetal bovine serum (FBS) and 50 µg/mL of gentamicin. Cells were cultured until 80% confluence for further analyses.

### 4.3. RNA Extraction and Reverse Transcription

Total RNAs from the tissue samples and cell lines were extracted using Trizol Reagent (Invitrogen, Carlsbad, CA, USA), following the manufacturer’s instructions. The RNA concentration and quality were analyzed in 1.5% agarose gel stained with GelRed 1X (Uniscience, Sao Paulo, Brazil), and spectrophotometrically by absorbance readings at 260 and 280 nm.

Reverse transcription was performed while using 1 μg of total RNA, 10 U RNaseIN (Invitrogen), 40 U of MMLV-RT (Invitrogen), 1X MMLV-RT buffer (Invitrogen), 200 μM of dNTPs (dGTP, dATP, dTTP and dCTP), and 126 pmols of random hexamers (Invitrogen). The reactions were performed at 37 °C for 1 h, followed by a denaturation step at 95 °C for 5 min.

### 4.4. Quantification of PBRM1 Transcripts

Real-time PCR was performed while using an ABI PRISM 7300 Sequence Detection System (Applied Biosystems, Carlsbad, CA, USA), and primers were designed for *PBRM1* gene (5′-CCATATCACTACAACAGATCCGAAC-3′, and 5′-ATATCGTCTCTCCTGGCAAGC-3′) and for *TGF-β* gene (5′-GTACCTGAACCCGTGTTGCTC-3′, and 5′- CAGGAATTGTTGCTGTATTTCTGG-3′). *Beta-2-microglobulina* (*B2M)* gene served as a reference gene for relative quantification and the primers were published previously [60]. The mRNA levels of *B2M* were not significantly altered across our groups, making it suitable as a housekeeper gene.

Each sample was tested in triplicate in a reaction containing 5.0 µL of Power SYBR Green PCR Master Mix (Applied Biosystems), and 0.5 µM of primers. Standard relative curves were performed to validate the 2^−ΔΔCq comparative method.

### 4.5. Immunohistochemistry

Tissue microarrays (TMAs) of 66 PCa tissues were used for immunohistochemistry. The patients’ ages ranged from 44 to 78 years (mean: 60.97 years ± 8.23). The average of PSA serum levels before surgery was 6.3 ng/mL (± 5.33 ranging from 0 to 24 ng/mL) and post-surgery was 0.16 ng/mL (± 0.71 ranging from 0 to 4.64). Based on Gleason histological classification, the patients were categorized in the low Gleason group, when displaying a Gleason score < 7 (45.5%) and in the high Gleason group when displaying a Gleason score ≥ 7 (55.5%).

Immunohistochemistry reactions were carried out by the following steps: incubation with citrate buffer 6 M for 1 h at 90 °C for antigen retrieval; peroxidase blockage with H_2_O_2_ 3% in water for 30 min., followed by blockage of unspecified sites with PBS/BSA 10 % for 1 h at room temperature. The slides were incubated for 16 h with anti-PBRM1 (Sigma, AMAb90690, St. Louis, MO, USA) at 1:25 dilution. Next, the slides were incubated with Novolink™ Max Polymer Detection System (Leica Biosystems, Newcastle, UK), revealed with diaminobenzidine substrate solution (DAB, Sigma), and counterstained with hematoxylin. The control sections were only incubated with PBS. The photomicrographs were made by the software HLImage (Western Vision Software, Salt Lake City, UT, USA), and nuclear, membrane, and cytoplasmic staining of PBRM1 were analyzed by a pathologist blinded to patient information.

### 4.6. PBRM1 Knockdown

Lentiviral particles encoding a scrambled shRNA sequence (1.0 × 10^5^ IFU; shControl, sc-108080, Santa Cruz Biotechnology, Dallas, TX, USA) or a pool of lentiviral particles encoding four shRNAs specific for PBRM1 knockdown (1.0 × 10^5^ IFU; shPBRM1, sc-76075-V, Santa Cruz Biotechnology) were used. The PC-3 cells were previously incubated with Polybrene at 5 μg/mL (Santa Cruz Biotechnology) and subsequently transduced with the viral particles. Clones expressing the shPBRM1 were selected by using 5 μg/mL of puromycin as a selective agent (Santa Cruz Biotechnology).

### 4.7. Cell Lysis and Western Blotting

The nuclear and cytoplasmic protein extracts were obtained from 5 × 10^5^ RWPE-1, LNCaP, DU-145, and PC-3 cells, by the aid of NE-PER Nuclear and Cytoplasmic Extraction Reagent kit (Thermoscientific, Dallas, TX, USA), following the manufacturer’s instructions. Protein concentration was measured by BCA (BCA™ Protein Assay; Thermo Scientific™ Pierce™, Dallas, TX, USA).

The proteins were separated in a 10% polyacrylamide gel and transferred onto nitrocellulose membrane (Hybond, GE Healthcare, Chicago, IL, USA). Membranes were blocked for 1h with PBS-5% milk and subsequently incubated with anti-PBRM1 (rabbit polyclonal, 1:1000, A301-591A Bethyl, Montgomery, TX, USA), anti-Actin (mouse monoclonal; 1:3000; AC-74, Sigma), and anti-Lamin B2 (mouse monoclonal; 1:2000; SAB2702205, Sigma). Finally, HRP-conjugated goat anti-rabbit IgG (1:1000, Thermoscientific, G21234) was used and the proteins were detected by chemioluminescence (ECL detection reagents^®^—GE HealthCare) on autoradiographic films (GE HealthCare).

### 4.8. Immunofluorescence

RWPE-1, LNCaP, DU-145, and PC-3 cells were fixed with 3 % paraformaldehyde and permeabilized by using PBS 0,1 % Triton. The cells were incubated with anti-PBRM1 antibody (1:50, Sigma, AMAb90690) and subsequently with a FITC-conjugated goat anti-mouse IgG (1:100, Sigma, F0257). The nuclei were counterstained with TO-PRO-3^®^ Iodide (1:500, Invitrogen). Cell staining was analyzed by confocal microscopy (*Zeiss LSM510*, Carl Zeiss AG, Oberkochen, Germany).

### 4.9. Flow Cytometry

The cells were fixed and permeabilized by Cyto Fix/Cyto Perm kit to verify the level of EMT and CSC markers (BD PharmingenTM, San Jose, CA, USA). Subsequently, the cells were incubated with anti-EpCAM-APC (5:100; 324208, Biolegend) and anti-Vimentin-Alexa Flour 488 (5:100; 562338, BD Biosciences, San Jose, CA, USA). Cells were also stained with anti-N-Cadherin (1:100; 333900, Thermo Fisher, Waltham, MA, USA), followed by incubation with PE-conjugated goat anti-mouse IgG (1:100; M30004-1, Invitrogen), and with anti-E-Cadherin (1:100, 491009, Invitrogen), followed by incubation with FITC-conjugated goat anti-rabbit IgG (1:100; 656111, Invitrogen). As controls, the cells were stained with APC Mouse IgG2b, κ (5:100; 400322, Biolegend, San Diego, CA, USA), with Alexa Fluor 488 Mouse IgG1 κ (5:100; 557721, BD Biosciences), with PE-conjugated goat anti-mouse IgG (1:100; M30004-1, Invitrogen) or with FITC-conjugated goat anti-rabbit IgG (1:100; 656111, Invitrogen). Flow Cytometry analyzed the cells (Accuri C6, BD Pharmingen™).

### 4.10. Wound Healing Assay

Cell migration was measured in a classical wound healing assay [61]. The assay was performed in triplicate by comparing the PC-3 shControl and PC-3 shPBRM1 cells. A total of 3 × 10^6^ cells/well were seeded onto six-well plates. Pictures of the wounds (10×) were taken at times 0 and 24 h in serum-free medium (EVOS^®^, AMG).

### 4.11. Statistical Analysis

The statistical analyses were performed using the software GraphPad Prism 7.0 (GraphPad Software Inc., La Jolla, CA, USA). The differences in transcriptional levels were evaluated by using Mann–Whitney test and χ^2^ test in order to verify the efficacy in discriminating PCa vs BPH groups. To verify the consistency in response across two variables, it was established a cut-off value for Odds ratio (OR) analysis. For protein staining data, the χ^2^ test was used to evaluate the significance. The results that were obtained for cell lines were analyzed by using the Student’s T-test. Statistical significance was considered when *p* < 0.05.

## 5. Conclusions

Taken together, our results corroborate the association of PBRM1 with PCa progression. High transcription and translation levels of PBRM1 were associated to higher Gleason score. Moreover, we showed that PBRM1 displays tumor-progression activity in PCa by enhancing the EMT process and the expression of CSC markers. Therefore, our data supported the hypothesis that the PBRM1, which is a unique component of the PBAF complex, is important in prostate malignant transformation and aggressiveness.

## Figures and Tables

**Table 1:** Patients’ characteristics. Comparison of clinical and biochemical parameters were conducted between prostate cancer (PCa) (N = 27) and benign prostatic hyperplasia (BPH) patients (N = 13). The results indicated that patients’ age and Prostate-Specific Antigen (PSA) levels did not differ between both groups.

Characteristics	PCa Patients	BPH Patients	*p*-value
Age (years)			
Mean ± SD	65.6 ± 6.1	70.9 ± 8.2	
Median (Range)	62 (51–77)	71 (59–87)	
<65	11 (41)	4 (31)	0.54 ^a^
≥65	16 (59)	9 (69)	
PSA (ng/mL)			
Mean ± SD	9.57 ± 6.77	8.75 ± 6.27	
Median (Range)	7.88 (2.16–33.1)	8.4 (2.5–24.28)	
<4	5 (18)	5 (38)	0.25 ^a^
≥4	22 (82)	8 (62)	
<10	17 (63)	8 (62)	0.47 ^a^
≥10	10 (37)	5 (38)	

^a^ Chi-square test of PCa × BPH patients; PSA: Prostate-Specific Antigen.

**Table 2:** Comparison of the transcriptional levels of *PBRM1* gene in PCa and BPH patients categorized according to their clinical and biochemical data. Odds ratio was calculated for each parameter among subjects classified as expressing low/high mRNA levels of *PBRM1* based on median value of mRNA levels. The results for Gleason score were significant.

**mRNA Expression of *PBRM1* in PCa Patients**					
	**N (%)**	***p*-value ^a^**	**Odds Ratio**		
Age (years)			**Low N (%)**	**High N (%)**	***p*-value**
<65	11 (41)	0.19	5 (18)	6 (22)	0.71
≥65	16 (59)		9 (34)	7 (26)	
			0.65 (95% CI:0.14–3.05)		
PSA (ng/mL)					
<4	5 (18)	0.05	4 (15)	1 (3)	0.33
≥4	22 (82)		10 (37)	12 (45)	
			4.8 (95% CI:0.56–6285)		
<10	17 (63)	0.18	10 (37)	7 (26)	0.44
≥10	10 (37)		4 (15)	6 (22)	
			2.14 (95% CI:0.43–8.80)		
Gleason score					
<7	13 (48)	0.04 *	10 (37)	3 (11)	0.02 *
≥7	14 (52)		4 (15)	10 (37)	
			8.33 (95% CI:1.55–36.94)		
**mRNA Expression of *PBRM1* in BPH Patients**					
	**N (%)**	***p*-value**	**Odds Ratio**		
Age (years)			**Low N (%)**	**High N (%)**	***p*-value**
<65	4	0.46	3	1	0.99
≥65	9		6	3	
			1.5 (95% CI:0.14–25.3)		
PSA (ng/mL)					
<4	5	0.39	5	1	0.58
≥4	8		5	3	
			3.0 (95% CI:0.31–45.7)		
<10	8	0.39	5	3	0.99
≥10	5		3	2	
			1.1 (95% CI:0.33–8.47)		

^a^ Mann-Whitney test of mRNA levels of *PBRM1* in PCa patients; * *p* < 0.05 is significant; PSA: Prostate-Specific Antigen. 95% CI: 95% confidence interval.

## Abbreviations

B2M	Beta-2 microglobulin
BAF180	Polybromo-1
Bh	Bromo-adjacent homology domains
BPH	Benign prostatic hyperplasia
BrDs	Bromodomains
CRPC	Castration-Resistant Prostate Cancer
Hmg	High-mobility group
IC	Confidence interval
OR	Odds Ratio
PBRM1	Polybromo-1
PCa	Prostate Cancer
PSA	Prostate-specific antigen
SWI/SNF	Switch/Sucrose Non-Fermentable
SWI/SNFB or PBAF	Polybromo-associated BAF
TNM	Tumor-Node-Metastasis

## References

[1] The Lancet Oncology (2018). New interventions offer prostate cancer hope. Lancet Oncol..

[2] Wyatt A.W., Mo F., Wang Y., Collins C.C. (2013). The diverse heterogeneity of molecular alterations in prostate cancer identified through next-generation sequencing. Asian J. Androl..

[3] Perlmutter M.A., Lepor H. (2007). Androgen deprivation therapy in the treatment of advanced prostate cancer. Rev. Urol..

[4] Roach M. (2014). Current trends for the use of androgen deprivation therapy in conjunction with radiotherapy for patients with unfavorable intermediate-risk, high-risk, localized, and locally advanced prostate cancer. Cancer.

[5] Montgomery R.B., Mostaghel E.A., Vessella R., Hess D.L., Kalhorn T.F., Higano C.S., True L.D., Nelson P.S. (2008). Maintenance of intratumoral androgens in metastatic prostate cancer: A mechanism for castration-resistant tumor growth. Cancer Res..

[6] Sridhar S.S., Freedland S.J., Gleave M.E., Higano C., Mulders P., Parker C., Sartor O., Saad F. (2014). Castration-resistant prostate cancer: From new pathophysiology to new treatment. Eur. Urol..

[7] Grant C.M., Kyprianou N. (2013). Epithelial mesenchymal transition (EMT) in prostate growth and tumor progression. Transl. Androl. Urol..

[8] Li P., Yang R., Gao W.Q. (2014). Contributions of epithelial-mesenchymal transition and cancer stem cells to the development of castration resistance of prostate cancer. Mol. Cancer.

[9] Shibue T., Weinberg R.A. (2017). EMT, CSCs, and drug resistance: The mechanistic link and clinical implications. Nat. Rev. Clin. Oncol..

[10] Sharma S., Zapatero-Rodriguez J., O’Kennedy R. (2017). Prostate cancer diagnostics: Clinical challenges and the ongoing need for disruptive and effective diagnostic tools. Biotechnol Adv..

[11] Kadoch C., Crabtree G.R. (2015). Mammalian SWI/SNF chromatin remodeling complexes and cancer: Mechanistic insights gained from human genomics. Sci. Adv..

[12] Lu C., Allis C.D. (2017). SWI/SNF complex in cancer. Nat. Genet..

[13] Hodges C., Kirkland J.G., Crabtree G.R. (2016). The Many Roles of BAF (mSWI/SNF) and PBAF Complexes in Cancer. Cold Spring Harbor Perspect. Med..

[14] Wilson B.G., Roberts C.W. (2011). SWI/SNF nucleosome remodellers and cancer. Nat. Rev. Cancer.

[15] Wu J.N., Roberts C.W. (2013). ARID1A mutations in cancer: Another epigenetic tumor suppressor?. Cancer Discov..

[16] Oike T., Ogiwara H., Nakano T., Yokota J., Kohno T. (2013). Inactivating mutations in SWI/SNF chromatin remodeling genes in human cancer. Jpn. J. Clin. Oncol..

[17] Thompson M. (2009). Polybromo-1: The chromatin targeting subunit of the PBAF complex. Biochimie.

[18] Wang H., Qu Y., Dai B., Zhu Y., Shi G., Zhu Y., Shen Y., Zhang H., Ye D. (2017). PBRM1 regulates proliferation and the cell cycle in renal cell carcinoma through a chemokine/chemokine receptor interaction pathway. PLoS ONE.

[19] Xia W., Nagase S., Montia A.G., Kalachikov S.M., Keniry M., Su T., Memeo L., Hibshoosh H., Parsons R. (2008). BAF180 is a critical regulator of p21 induction and a tumor suppressor mutated in breast cancer. Cancer Res..

[20] Brownlee P.M., Chambers A.L., Cloney R., Bianchi A., Downs J.A. (2014). BAF180 promotes cohesion and prevents genome instability and aneuploidy. Cell Rep..

[21] Lee H., Dai F., Zhuang L., Xiao Z.D., Kim J., Zhang Y., Ma L., You M.J., Wang Z., Gan B. (2016). BAF180 regulates cellular senescence and hematopoietic stem cell homeostasis through p21. Oncotarget.

[22] Burrows A.E., Smogorzewska A., Elledge S.J. (2010). Polybromo-associated BRG1-associated factor components BRD7 and BAF180 are critical regulators of p53 required for induction of replicative senescence. Proc. Natl. Acad. Sci. USA.

[23] Niimi A., Hopkins S.R., Downs J.A., Masutani C. (2015). The BAH domain of BAF180 is required for PCNA ubiquitination. Mutat. Res..

[24] Hopson S., Thompson M.J. (2017). BAF180: Its Roles in DNA Repair and Consequences in Cancer. ACS Chem. Biol..

[25] Varela I., Tarpey P., Raine K., Huang D., Ong C.K., Stephens P., Davies H., Jones D., Lin M.L., Teague J. (2011). Exome sequencing identifies frequent mutation of the SWI/SNF complex gene PBRM1 in renal carcinoma. Nature.

[26] Huang L., Peng Y., Zhong G., Xie W., Dong W., Wang B., Chen X., Gu P., He W., Wu S. (2015). PBRM1 suppresses bladder cancer by cyclin B1 induced cell cycle arrest. Oncotarget.

[27] Shu X.S., Zhao Y., Sun Y., Zhong L., Cheng Y., Zhang Y., Ning K., Tao Q., Wang Y., Ying Y. (2018). The epigenetic modifier PBRM1 restricts the basal activity of the innate immune system by repressing retinoic acid-inducible gene-I-like receptor signalling and is a potential prognostic biomarker for colon cancer. J. Pathol..

[28] Sekine I., Sato M., Sunaga N., Toyooka S., Peyton M., Parsons R., Wang W., Gazdar A.F., Minna J.D. (2005). The 3p21 candidate tumor suppressor gene BAF180 is normally expressed in human lung cancer. Oncogene.

[29] Link K.A., Balasubramaniam S., Sharma A., Comstock C.E., Godoy-Tundidor S., Powers N., Cao K.H., Haelens A., Claessens F., Revelo M.P. (2008). Targeting the BAF57 SWI/SNF subunit in prostate cancer: A novel platform to control androgen receptor activity. Cancer Res..

[30] Shen H., Powers N., Saini N., Comstock C.E., Sharma A., Weaver K., Revelo M.P., Gerald W., Williams E., Jessen W.J. (2008). The SWI/SNF ATPase Brm is a gatekeeper of proliferative control in prostate cancer. Cancer Res..

[31] Liu X.B., Sun A.J., Wang C., Chen L.R. (2010). Expression of BRG1 and BRM proteins in prostatic cancer. Zhonghua Bing Li Xue Za Zhi.

[32] Hansen R.L., Heeboll S., Ottosen P.D., Dyrskjot L., Borre M. (2011). Smarcc1 expression: A significant predictor of disease-specific survival in patients with clinically localized prostate cancer treated with no intention to cure. Scand. J. Urol. Nephrol..

[33] Prensner J.R., Iyer M.K., Sahu A., Asangani I.A., Cao Q., Patel L., Vergara I.A., Davicioni E., Erho N., Ghadessi M. (2013). The long noncoding RNA SChLAP1 promotes aggressive prostate cancer and antagonizes the SWI/SNF complex. Nat. Genet..

[34] Andriole G.L., Catalona W.J. (1993). Using PSA to screen for prostate cancer. The Washington University experience. Urol. Clin. N. Am..

[35] Nogueira L., Corradi R., Eastham J.A. (2009). Prostatic specific antigen for prostate cancer detection. Int. Braz J. Urol..

[36] Xu S., Zhan M., Wang J. (2017). Epithelial-to-mesenchymal transition in gallbladder cancer: From clinical evidence to cellular regulatory networks. Cell Death Discov..

[37] Ni J., Cozzi P., Hao J., Beretov J., Chang L., Duan W., Shigdar S., Delprado W., Graham P., Bucci J. (2013). Epithelial cell adhesion molecule (EpCAM) is associated with prostate cancer metastasis and chemo/radioresistance via the PI3K/Akt/mTOR signaling pathway. Int. J. Biochem. Cell Biol..

[38] Zavadil J., Bottinger E.P. (2005). TGF-beta and epithelial-to-mesenchymal transitions. Oncogene.

[39] Dong L., Zieren R.C., Xue W., de Reijke T.M., Pienta K.J. (2019). Metastatic prostate cancer remains incurable, why?. Asian J. Urol..

[40] Kounatidou E., Nakjang S., McCracken S.R.C., Dehm S.M., Robson C.N., Jones D., Gaughan L. (2019). A novel CRISPR-engineered prostate cancer cell line defines the AR-V transcriptome and identifies PARP inhibitor sensitivities. Nucl. Acids Res..

[41] Ellinger J., Kahl P., von der Gathen J., Rogenhofer S., Heukamp L.C., Gutgemann I., Walter B., Hofstadter F., Buttner R., Muller S.C. (2010). Global levels of histone modifications predict prostate cancer recurrence. Prostate.

[42] Thoma C. (2017). Prostate cancer: Epigenetic AR regulation. Nat. Rev. Urol..

[43] Langst G., Manelyte L. (2015). Chromatin Remodelers: From Function to Dysfunction. Genes.

[44] Skulte K.A., Phan L., Clark S.J., Taberlay P.C. (2014). Chromatin remodeler mutations in human cancers: Epigenetic implications. Epigenomics.

[45] Chowdhury B., Porter E.G., Stewart J.C., Ferreira C.R., Schipma M.J., Dykhuizen E.C. (2016). PBRM1 Regulates the Expression of Genes Involved in Metabolism and Cell Adhesion in Renal Clear Cell Carcinoma. PLoS ONE.

[46] da Costa W.H., Rezende M., Carneiro F.C., Rocha R.M., da Cunha I.W., Carraro D.M., Guimaraes G.C., de Cassio Zequi S. (2014). Polybromo-1 (PBRM1), a SWI/SNF complex subunit is a prognostic marker in clear cell renal cell carcinoma. BJU Int..

[47] Kim J.Y., Lee S.H., Moon K.C., Kwak C., Kim H.H., Keam B., Kim T.M., Heo D.S. (2015). The Impact of PBRM1 Expression as a Prognostic and Predictive Marker in Metastatic Renal Cell Carcinoma. J. Urol..

[48] Macher-Goeppinger S., Keith M., Tagscherer K.E., Singer S., Winkler J., Hofmann T.G., Pahernik S., Duensing S., Hohenfellner M., Kopitz J. (2015). PBRM1 (BAF180) protein is functionally regulated by p53-induced protein degradation in renal cell carcinomas. J. Pathol..

[49] Murakami A., Wang L., Kalhorn S., Schraml P., Rathmell W.K., Tan A.C., Nemenoff R., Stenmark K., Jiang B.H., Reyland M.E. (2017). Context-dependent role for chromatin remodeling component PBRM1/BAF180 in clear cell renal cell carcinoma. Oncogenesis.

[50] Pawlowski R., Muhl S.M., Sulser T., Krek W., Moch H., Schraml P. (2013). Loss of PBRM1 expression is associated with renal cell carcinoma progression. Int. J. Cancer.

[51] Mo D., Li C., Liang J., Shi Q., Su N., Luo S., Zeng T., Li X. (2015). Low PBRM1 identifies tumor progression and poor prognosis in breast cancer. Int. J. Clin. Exp. Pathol..

[52] Epstein J.I., Zelefsky M.J., Sjoberg D.D., Nelson J.B., Egevad L., Magi-Galluzzi C., Vickers A.J., Parwani A.V., Reuter V.E., Fine S.W. (2016). A Contemporary Prostate Cancer Grading System: A Validated Alternative to the Gleason Score. Eur. Urol..

[53] McGuire B.B., Helfand B.T., Loeb S., Hu Q., O’Brien D., Cooper P., Yang X., Catalona W.J. (2012). Outcomes in patients with Gleason score 8–10 prostate cancer: Relation to preoperative PSA level. BJU Int..

[54] Humphrey P.A. (2004). Gleason grading and prognostic factors in carcinoma of the prostate. Mod. Pathol..

[55] Zhu G.J., Song P.P., Zhou H., Shen X.H., Wang J.G., Ma X.F., Gu Y.J., Liu D.D., Feng A.N., Qian X.Y. (2018). Role of epithelial-mesenchymal transition markers E-cadherin, N-cadherin, beta-catenin and ZEB2 in laryngeal squamous cell carcinoma. Oncol. Lett..

[56] Tanaka H., Kono E., Tran C.P., Miyazaki H., Yamashiro J., Shimomura T., Fazli L., Wada R., Huang J., Vessella R.L. (2010). Monoclonal antibody targeting of N-cadherin inhibits prostate cancer growth, metastasis and castration resistance. Nat. Med..

[57] Jennbacken K., Tesan T., Wang W., Gustavsson H., Damber J.E., Welen K. (2010). N-cadherin increases after androgen deprivation and is associated with metastasis in prostate cancer. Endocr. Relat. Cancer.

[58] Collazo J., Zhu B., Larkin S., Martin S.K., Pu H., Horbinski C., Koochekpour S., Kyprianou N. (2014). Cofilin drives cell-invasive and metastatic responses to TGF-beta in prostate cancer. Cancer Res..

[59] Karan D., Kelly D.L., Rizzino A., Lin M.F., Batra S.K. (2002). Expression profile of differentially-regulated genes during progression of androgen-independent growth in human prostate cancer cells. Carcinogenesis.

[60] Araujo T.G., Marangoni K., Rocha R.M., Maia Y.C., Araujo G.R., Alcantar T.M., Alves P.T., Calabria L., Neves A.F., Soares F.A. (2014). Dynamic dialog between cytokeratin 18 and annexin A1 in breast cancer: A transcriptional disequilibrium. Acta Histochem..

[61] Liang C.C., Park A.Y., Guan J.L. (2007). In vitro scratch assay: A convenient and inexpensive method for analysis of cell migration in vitro. Nat. Protoc..

